# Study on the Cooling Effect of Asphalt Pavement Blended with Composite Phase Change Materials

**DOI:** 10.3390/ma15093208

**Published:** 2022-04-29

**Authors:** Ming Dai, Shiwan Wang, Jianbo Deng, Zhijie Gao, Zhiyun Liu

**Affiliations:** 1 China Railway Construction Investment Group Co., Ltd., Urumqi 830017, China; daim1975@126.com (M.D.); dengjianbo1982@126.com (J.D.); gaozj18310996321@163.com (Z.G.); 2 Department of Safety Engineering, College of Geology Engineering and Geomatics, Chang’an University, Xi’an 710054, China; 2020126138@chd.edu.cn

**Keywords:** asphalt pavement, phase change materials, cooling effect, earth-atmosphere heat transfer model, high temperature frequency and duration

## Abstract

To explore the cooling effect of phase change materials (PCM) on asphalt pavement, a numerical model of the coupled heat transfer process of a typical monolithic subgrade of the G7 Expressway in the eastern Tianshan mountain area was developed. Three types of paraffin materials (OP55E, OP52E, OP47E) were mixed in a 4:3:3 volume ratio and blended into the asphalt upper layer and overall asphalt layer at volume ratios of 5%, 10%, 15% and 20%. The cooling effect of different PCM addition schemes was simulated and analyzed, and the frequency and duration of asphalt pavement high temperature operation status were also measured. The results showed that: (1) Th addition of PCM in the asphalt layer can effectively reduce the frequency of pavement high temperature rutting damage. The number of days and average daily duration of high temperature on the road surface were both reduced. (2) The cooling effect was positively correlated with the PCM volume mixing ratio, and the temperature drop of the pavement also increased with the increase of the PCM blending ratio. As the PCM mixing ratio increased from 5% to 20%, the initial 75 °C pavement cooled by 1.49 °C and 4.66 °C, respectively, and the number of days and hours of pavement temperature over 70 °C decreased to 4 days and 3.3 h, respectively. (3) The cooling effect of the asphalt upper layer PCM scheme was greater at a small mixing ratio (5%), whereas the performance of the overall asphalt layer PCM blended scheme was effectively promoted by increasing the equivalent heat capacity of system under the large mixing ratio (20%).

## 1. Introduction

The Beijing–Xinjiang expressway (G7), which started construction in 2012 with a total mileage of 2540 km, is an important corridor connecting Xinjiang with the mainland, and is also an important part of China’s “Belt and Road” initiative. Its opening to traffic is of great significance in promoting the economy of the eastern Tianshan mountain area in Xinjiang and ensuring national defense security. The Balikun–Wutong Daquan section of the G7 is a typical high temperature and solar radiation area, where the duration of sunshine long (average daily sunshine of 9.9 h in summer), and solar radiation is intense (average sunshine intensity of 703 W/m^2^ in summer). Coupled with the strong heat absorption of black asphalt pavement (absorption rate of 0.85–0.95), the pavement temperature can exceed 70 °C in the high temperature season [1]. Under the action of heavy traffic load and high temperature, the pavement is susceptible to rutting damage, thus seriously affecting the stability and durability of the road. Therefore, the problem of pavement high temperature needs to be solved urgently.

In recent years, phase change energy storage technology has gained widespread attention and rapid development as an effective solution capable of storing energy at high density in the form of latent heat of the phase change [2]. Research on asphalt pavement cooling technology has shown that the addition of PCM to highway asphalt pavement can effectively reduce the temperature of asphalt pavement, thus alleviating asphalt pavement rutting damage to a certain extent [3,4]. The latest research on PCM mainly includes the development and selection of PCM for roads, its temperature regulation performance, and its impact on the performance of asphalt and asphalt mixture. Currently, commonly used PCM materials for road applications include polyethylene glycol [2,3], paraffin [4], loaded composite PCM [5,6], and micro-encapsulated PCM [7,8].

Through indoor experiments adding PCM to asphalt mixture, it was found that the addition of PCM not only improved the heat storage capacity of asphalt mixture [9] and high temperature stability [10,11], but also enhanced the environmental adaptability of asphalt mixture [12,13]. Chen et al. [14] and He et al. [15] proposed the technical requirements for the application of PCM in asphalt mixtures and found that the addition of PCM had a significant cooling effect. Subsequently, researchers [16,17,18] summarized the mixing method of PCM in asphalt mixture and its corresponding temperature regulation effect. In addition to the above performance adjustment, the addition of PCM improves the heat storage and release performance of asphalt [19], increases the viscosity of asphalt [20], enhances the thermal conductivity and thermal diffusion coefficient of asphalt, and promotes rutting resistance ability of asphalt at high temperature [21]. Furthermore, the PCM-added asphalt mixture has good water stability and low temperature cracking performance [22]. In order to further study the role of PCM, researchers continued to explore the relationship between PCM content and the heat storage capacity of asphalt mixture. Zhou [23] and Cao et al. [24] measured the temperature regulation effect of PCM with different dosages on asphalt mixtures, proving that the temperature regulation effect was closely related to the initial temperature and PCM content. The study proves that the mixing ratio affects the temperature regulation, which paves the way for the further study of the cooling effect of PCM on asphalt pavement.

Under severe high temperature climatic conditions in summer, the asphalt pavement of Balikun–Wutong Daquan section of the expressway faces severe high-temperature rutting damage. The feasibility of pavement cooling technology for asphalt surface blending with PCM has been demonstrated by existing studies, but there is still a relative lack of research on the location of PCM addition and the overall temperature control effectiveness of pavements at an interannual scale. In this study, a coupled earth–atmosphere heat transfer numerical model containing the upper air environment was established for the integral roadbed section of the G7 expressway, and the meteorological data such as air temperature, wind speed and solar radiation in the area were used as external input conditions. The cooling effect of the asphalt pavement with different addition ratios and locations was compared and analyzed, and the frequency and duration of the high temperature range were also measured. The research results of this work can provide some reference for the application of PCM in the regulation of asphalt pavement temperature for the Gobi area.

## 2. Goals and Objectives

**Figure 1:**
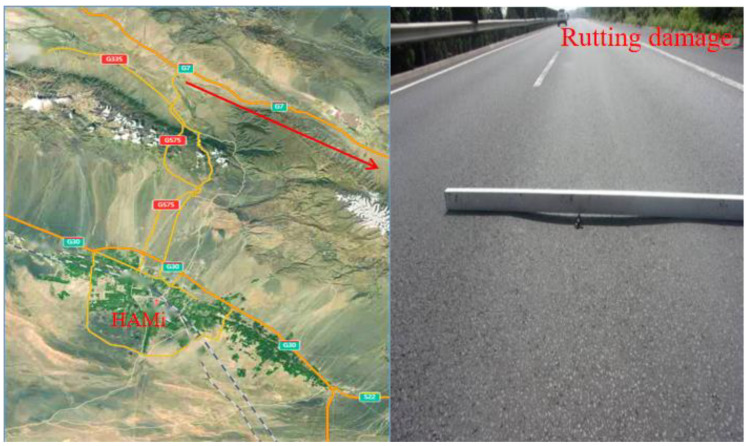
Typical rutting damage of highways in the Hami area.

Field survey showed that rutting damage on highways in the Hami area is frequent and serious (Figure 1). The triggers of rutting damage can be divided into internal factors and external factors [25,26]. Internal factors are usually related to the pavement structure and the quality of asphalt materials, whereas the external factors are mainly climatic and traffic conditions. Climatic conditions in the Hami area typically include high temperature and high solar radiation, which rapidly cause high temperatures of road surfaces and then lead to different degrees of rutting damage risk. According to literature research [27], the rutting damage risk level at different pavement temperature ranges is given in Table 1.

It has been demonstrated that adding PCM into asphalt mixtures can improve their environmental adaptability [12,13]. However, these studies are only short-term experimental or numerical simulation research, and there are still relatively few reports on the high temperature rutting risk of PCM-added asphalt pavement under the long-term influence of a high temperature climate. The research goal of this paper is to examine the long-term regulation effect of PCM on asphalt pavement temperature and obtain specific data for the frequency and duration of pavement high temperature. The numerical results can provide a basis for the prevention and maintenance of pavement damage in the high temperature Gobi area and also have important engineering significance and academic value.

## 3. Materials and Methods

### 3.1. Selection and Addition of PCM

#### 3.1.1. Selection of PCM

**Figure 2:**
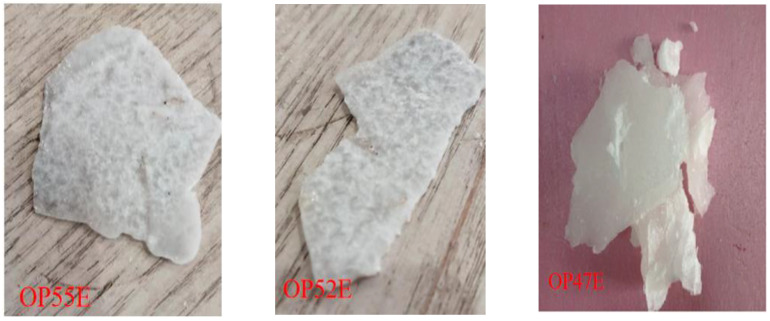
Pictures of different kinds of paraffin wax materials.

**Figure 3:**
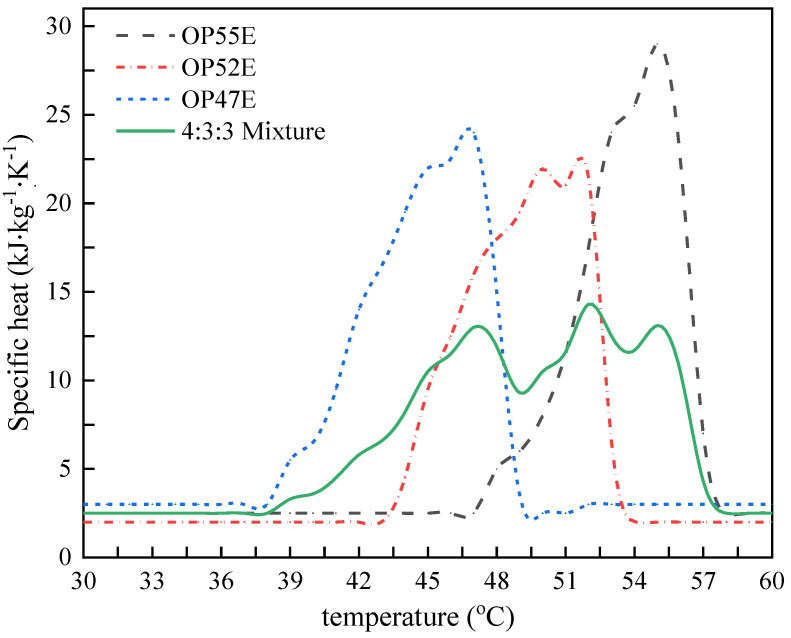
Specific heat of different phase change materials.

Selection of suitable PCM for asphalt pavements requires consideration of factors such as the temperature range of the asphalt pavement in high temperature climates and the materials used to lay the asphalt pavement [28]. According to literature data, the critical temperature for rutting damage on asphalt pavements was generally 55–60 °C [29]. Therefore, the phase change point of the PCM must be below 60 °C. In the screening of PCM for roads in recent years, paraffin wax (phase change interval of 47–64 °C, density of about 0.9 g/cm^3^) has been widely used for its advantages including low price, high latent heat, and convenient access. Thus, paraffin wax was also chosen as the PCM for the asphalt surface layer in this work. Additionally, to achieve a better cooling effect, the phase change temperature range of the mixed PCM in the asphalt surface layer should be as large as possible. By comparing the phase change interval of different types of paraffin wax materials, it was found that the phase change interval of a single type of paraffin wax material was relatively small. Therefore, to expand the phase change temperature interval, three types of paraffin wax materials, OP55E, OP52E, and OP47E (Provided by Hangzhou Ruer Energy Technology Co. LTD., Hangzhou, China, as shown in Figure 2), with phase change intervals of 51–57 °C, 49–53 °C, and 41–48 °C, respectively, were mixed. The thermal physical parameters of the three paraffin wax materials are detailed in Table 2. By adjusting the mixing ratio, it was concluded that when the volume mixing ratio of the three PCM was 4:3:3, the average specific heat capacity and its variance of the composite PCM obtained the maximum and minimum values, respectively. Therefore, the volume mixing ratio of the ternary composite PCM was defined as 4:3:3. The specific heat of PCM mixture was 11,810 J/(kg·K), and the phase change temperature interval of the composite PCM was 47–56 °C, as shown in Figure 3.

#### 3.1.2. Addition Schemes of PCM

The strong heat absorption of the black asphalt surface of an expressway leads to the continuous high temperatures of the pavement and rutting damage in summer. The analysis of the overall heat transfer process between the road and environment showed that the location and values of the high temperature of the asphalt surface layer were related to the addition ratio and the location of the composite PCM. [30]

Two schemes were set for studying the cooling effect of PCM addition location: (1) addition only in the upper 5 cm of medium-grained asphalt concrete; (2) addition overall in the 12 cm of the asphalt concrete layer. Since the addition ratio of PCM was necessarily related to the cooling of the pavement, the relationship between different ratios of PCM and the magnitude of pavement cooling was also the research goal of this work. The PCM blending ratios at each addition position were configured with a gradient of 5%, which were 5%, 10%, 15%, and 20%, respectively. Taking no addition as Scheme 0, the other specific schemes are detailed in Table 3.

### 3.2. Calculation Model

The temperature distribution of asphalt pavement with different PCM addition schemes was studied by establishing a coupled earth-atmosphere heat transfer calculation model. The physical model of subgrade was chosen from a typical integral road section of the G7 Expressway. Its subsoil layers were a 0.7 m thick silt layer, 5 m thick gravel layer, and underneath undrilled sand layer. Assuming that the deep soil layer had little effect on the heat transfer process of the pavement, the total underground depth was taken as 30 m for calculation convenience.

**Figure 4:**
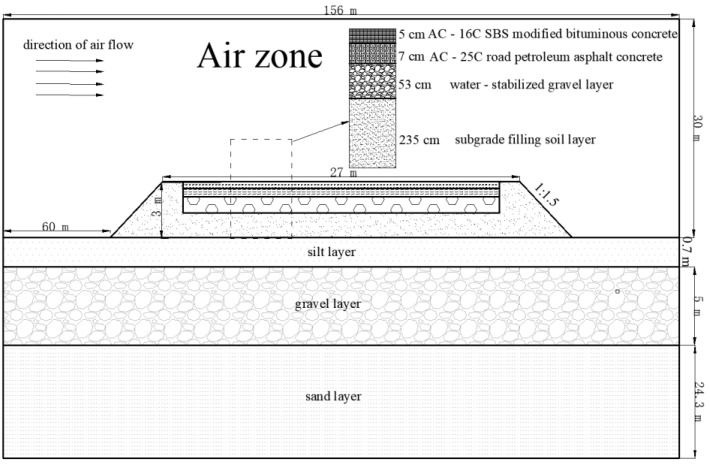
Schematic diagram of the road model.

The integral roadbed pavement, with a width of 27 m, a height of 3 m, and a 1:1.5 slope ratio, was composed of roadbed fill, a 53 cm water-stabilized gravel layer, and a 12 cm asphalt layer in order from the bottom to the top (5 cm AC-16C SBS modified bituminous concrete and 7 cm AC-25C road petroleum asphalt concrete). There were 0.75 m soil shoulders on the left and right of the road, with the same soil properties as the roadbed fill. The height of natural ground surface was taken as 20 times the height of roadbed, that is, 60 m on the left and right to avoid the entrance effect. The total height of air area was taken as 30 m to reduce the influence of upper boundary conditions. A schematic diagram of the road model is shown in Figure 4.

#### 3.2.1. Control Equations for Numerical Calculation

##### Air Zone

The air environment above the roadbed and natural ground surface was considered as a free fluid, and the air was considered as an incompressible gas with constant density. The control equations were listed as follows [31]:

Continuity Equation (1):

(1) $$\frac{\partial }{\partial x_{i}}\left(\rho u_{i}\right)=0,$$

where ρ is the air density, and $u_{i}\;$ is the velocity component in each direction.

Momentum Equation (2):

(2) $$\frac{\partial \left(\rho u_{i}\right)}{\partial t}+\frac{\partial }{\partial x_{i}}\left(\rho u_{i}u_{j}\right)=-\frac{\partial p}{\partial x_{i}}+\frac{\partial }{\partial x_{i}}\left(\eta \frac{\partial u_{i}}{\partial x_{i}}\right),$$

where p is the air pressure, and η is the dynamic viscosity of the air.

Energy Equation (3):

(3) $$\frac{\partial \left(\rho T\right)}{\partial t}+\frac{\partial }{\partial x_{i}}\left(\rho u_{i}T\right)=\frac{\partial }{\partial x_{i}}\left(\frac{\lambda }{c_{p}}\frac{\partial u_{i}}{\partial x_{i}}\right)+S_{T},$$

where *T* is the air temperature, λ  is the thermal conductivity of air, $c_{p}\;$ is the specific heat capacity of air at constant pressure, and $S_{T}\;$ is the coupled source term of natural ground surface and pavement.

κ  Equation (4):

(4) $$\frac{\partial \left(\rho \kappa \right)}{\partial t}+\frac{\partial }{\partial x_{i}}\left(\rho u_{i}\kappa \right)=\frac{\partial }{\partial x_{i}}\left[\left(\eta +\frac{\eta_{t}}{\sigma_{\kappa }}\right)\frac{\partial \kappa }{\partial x_{i}}\right]+G_{\kappa }-\rho \varepsilon ,$$

where κ  is the turbulent fluctuating kinetic energy, $\eta_{t}\;$ is the dynamic viscosity caused by turbulent fluctuation, $\sigma_{\kappa }\;$ is the Pr value of turbulence kinetic energy, $G_{\kappa }\;$ is the generation term of turbulent fluctuating kinetic energy, and ε  is the turbulence dissipation rate.

ε  Equation (5):

(5) $$\frac{\partial \left(\rho \varepsilon \right)}{\partial t}+\frac{\partial }{\partial x_{i}}\left(\rho u_{i}\varepsilon \right)=\frac{\partial }{\partial x_{i}}\left[\left(\eta +\frac{\eta_{t}}{\sigma_{\varepsilon }}\right)\frac{\partial \varepsilon }{\partial x_{i}}\right]+\frac{\varepsilon }{\lambda }\left(c_{1}G_{\kappa }-c_{2}\rho \varepsilon \right),$$

where $\sigma_{\varepsilon }\;$ is the Pr value of turbulent dissipation, and $c_{1}$ and $c_{2}$ are empirical constants.

##### Solid Zone

The solid region includes the pavement, embankment and the underlying active soil layer, and their main heat transfer mode is heat conduction. In this study, the equivalent heat capacity method was adopted in the heat transfer numerical calculation. The control equation was [32]:

(6) $$C^{*}\frac{\partial T}{\partial t}=\frac{\partial }{\partial x}\left(\lambda^{*}\frac{\partial T}{\partial x}\right)+\frac{\partial }{\partial y}\left(\lambda^{*}\frac{\partial T}{\partial y}\right),$$

where $C^{*}\;$ and $\lambda^{*}\;$ are the equivalent volumetric heat capacity and equivalent thermal conductivity of each solid material layer, respectively.

#### 3.2.2. Thermo-Physical Parameters

The physical properties of air in the fluid region were set as fixed values in numerical calculations. Referring to the actual regional climate measurements, the thermal parameters of air were specified as follows: ρ=1.225 kg/m^3^; the specific heat $C_{p}=1006$ J/(kg·K); thermal conductivity *λ* = 0.0242 W/(m·K); and air viscosity *μ* = 1.789 × 10^−5^ kg/(m·s). The thermophysical parameters of solid materials were all defined as standard measurement values, as shown in Table 4.

In the simulation, PCM and asphalt material of pavement were mixed in different proportions. The thermal properties of the mixture were calculated with reference to the empirical formula proposed by Su et al. [33]:

(7) $$C_{pm}=\frac{\phi \rho_{1}C_{p1}+\left(1-\phi \right)\rho_{2}C_{p2}}{\phi \rho_{1}+\left(1-\phi \right)\rho_{2}},$$

(8) $$\rho_{m}=\phi \rho_{1}+\left(1-\phi \right)\rho_{2},$$

(9) $$\lambda_{m}=\rho_{m}C_{pm}\frac{\phi \lambda_{1}+\left(1-\phi \right)\lambda_{2}}{\phi \rho_{1}C_{p1}+\left(1-\phi \right)\rho_{2}C_{p2}},$$

where $C_{pm}$ is the specific heat capacity of the mixture of asphalt and PCM, $\rho_{m}$ is the density of the mixture, $\lambda_{m}$ is the thermal conductivity of the mixture, ϕ is the volume ratio of added PCM, $\rho_{1}$ is the density of PCM, $C_{p1}$ is the specific heat capacity of PCM, $\lambda_{1}$ is the thermal conductivity of PCM, $\rho_{2}$ is the density of asphalt material, $C_{p2}$ is the specific heat capacity of asphalt material, and $\lambda_{2}$ is the thermal conductivity of the asphalt material.

#### 3.2.3. Boundary Conditions

**Figure 5:**
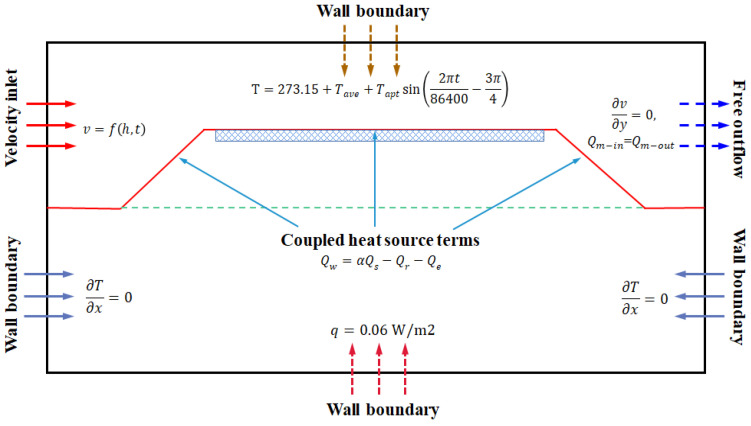
Schematic diagram of the boundary conditions.

In this study, the influential conditions including solar radiation, temperature, and wind speed in the Hami area were incorporated into the research model. The calculation model was an open system coupled with an ambient air environment, as shown in Figure 5. The setting methods of each boundary condition in the model were as follows: (I), the left side of the numerical model was defined as the velocity inlet boundary condition, in which temperature and wind speed varied over time; (II), the right side was adopted as the free outflow boundary conditions; (III), the upper surface of the model was defined as temperature variation wall condition; (IV), the left and right sides of the solid region were defined as adiabatic boundary conditions; (V), the bottom surface was chosen as the heat flux boundary with the specific value of 0.06 W/m^2^ [34]; (VI), Asphalt pavement, slope and natural ground surface were determined as coupled heat source terms considering for the combined effects of solar radiation, forced convection by wind, and surface evaporation. The solar radiation intensity, evaporation, air temperature, and wind speed data were all taken from the China meteorological data network (http://data.cma.cn) (accessed on 25 May 2021). The detailed calculation methods of air temperature, wind speed and coupled source terms were given as:

##### Air Temperature

The ambient air temperature was assumed as the form of a sine function in the numerical calculation:

(10) $$T=273.15+T_{ave}+T_{apt}\sin\left(\frac{2\pi t}{86400}-\frac{3\pi }{4}\right),$$

where $T_{ave}$ is the daily average air temperature, $T_{apt}$ is the daily air temperature amplitude. $T_{ave}$ and $T_{apt}$ are obtained from statistical data of temperature for the Hami area between 1 May and 30 September.

##### Surface Source Term

Through the analysis of the coupled heat transfer process between the surface and upper air environment, natural ground surface, slope surface, and road surface were set as a coupled wall boundary condition with a thickness of 0.1 m [35]. The specific value of source term $Q_{w}$, which was the net energy budget of the surface, can be obtained as follows:

(11) $$Q_{w}=\alpha Q_{s}-Q_{r}-Q_{e},$$

where α is the absorption coefficient which is taken as a constant value on different surfaces (0.65, 0.72, and 0.9 for natural ground, slope, and pavement, respectively), $Q_{s}$ is the total solar radiation received, $Q_{r}$ is the long wave radiation heat loss to the environment, and $Q_{e}$ is the heat taken away by evaporation of water. The calculation methods of the relevant parameters were detailed in the reference [35].

##### Wind Speed and Direction

Account for the main wind direction in the Hami area, the wind direction was taken as entering vertically from the left velocity inlet boundary and outflowing from the right side of the computation model. And the wind speed was taken as the daily average wind speed based on the meteorological database. Furthermore, the wind speed was also set as the function of the height from the ground:

(12) $$v=v_{r}{\left(\frac{h}{h_{r}}\right)}^{0.14}$$

where h is the daily average wind speed of reference height, $h_{r}$ is the height reference spot and chosen as 3 m in this work.

#### 3.2.4. Calculation Method

**Figure 6:**
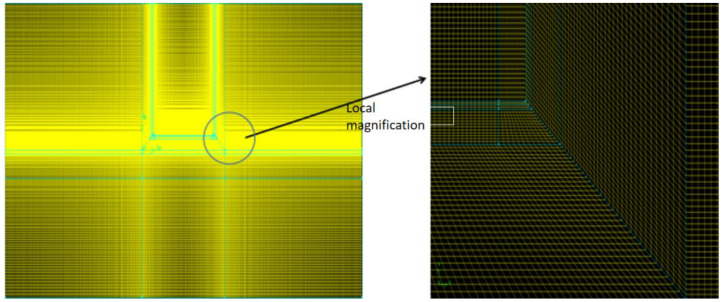
Computation grid of the numerical model.

The GAMBIT 2.4 software was used to discretize the mesh with the dissected quadrilateral structure. The critical coupling heat transfer areas such as the flow field area with dramatic change and the studied roadbed area were encrypted, with the mesh aspect ratio less than 1:2 and the mesh ratio of non-critical areas not higher than 1:10. Some of the details are shown in Figure 6. The earth-atmosphere coupled numerical model was adopted in this work, which had been validated by comparing numerical calculation results with field monitoring data in our previous published paper [36,37]. The control equations for earth-atmosphere heat transfer processes were solved in the ANSYS Fluent software package. Two-dimensional, unsteady and implicit solver was adopted in the numerical calculation. The standard κ-ε model was applied in the turbulence simulation of the air region. The complex boundary conditions of air temperature, wind speed and source term of surface were imported into the calculation model by Fluent UDF program. In addition, the convergence conditions during calculation were set as 10^−5^. To better reflect the real-time temperature, the calculation time step was selected as 20 min, and the total calculation time was defined as 153 days.

## 4. Results

### 4.1. Cooling Efficiency of Asphalt Pavement Mixed with PCM

**Figure 7:**
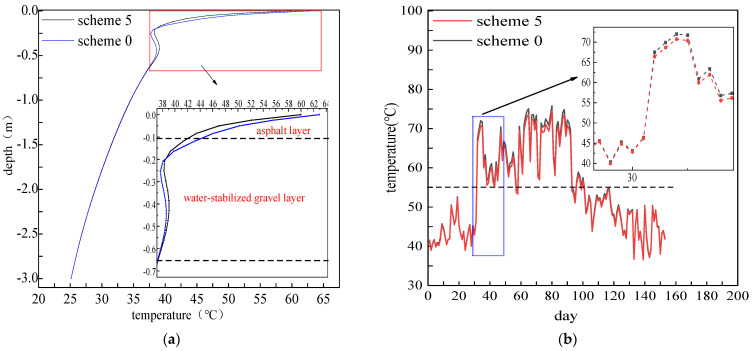
Temperature distributions of the pavement structure layer with different PCM mixing schemes: (**a**) middle hole temperature distributions; (**b**) daily maximum pavement temperature between 1 May to 30 September.

PCM with a volume ratio of 5% was added to a 12 cm asphalt layer to explore the cooling effect of PCM on asphalt pavement. Figure 7 shows the temperature distributions of the pavement structure layer with different PCM mixing schemes (non-PCM blended and overall asphalt layer PCM blended) at 12:00 noon on 15 July 2020. As shown in Figure 7a, the main area of drastic temperature changes is concentrated on the asphalt layer (−0.2–0 m), which has a temperature variation of nearly 30 °C. After mixing PCM in the asphalt upper layer, its temperature dropped significantly. By comparing the daily pavement maximum temperature from 1 May to 30 September, as shown in Figure 7b, it was found that the pavement maximum temperature is generally above 50 °C in June and July and the PCM adding scheme can effectively reduce the temperature, especially when the pavement temperature is higher than 55 °C.

**Figure 8:**
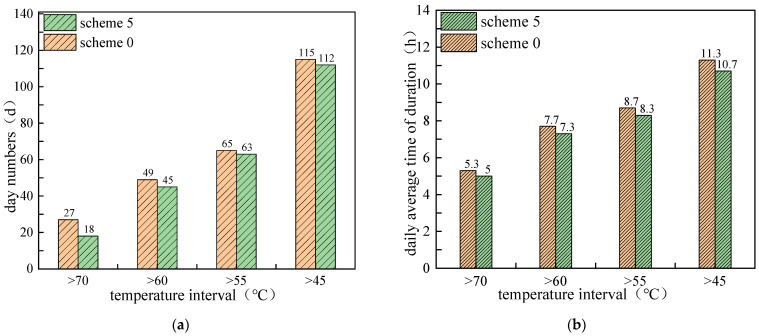
Duration times of daily maximum pavement temperature in different temperature ranges: (**a**) number of days; (**b**) Daily average duration in hours.

The duration times of daily maximum pavement temperature in different temperature ranges are given in Figure 8, which shows that, from 1 May to 30 September, the number of days and daily duration hours of all temperature ranges clearly decreased. After mixing PCM, the days of high temperature in the range of 45–55 °C, 55–60 °C, 60–70 °C and above 70 °C decreased by 9 days, 4 days, 2 days and 3 days, respectively, and daily average duration decreased by 0.3, 0.4, 0.4 and 0.6 h, respectively. It can be concluded that the addition of PCM in the asphalt layer can effectively reduce the frequency of pavement high temperature rutting damage.

### 4.2. Cooling Efficiency of Different Mixing Ratios

**Figure 9:**
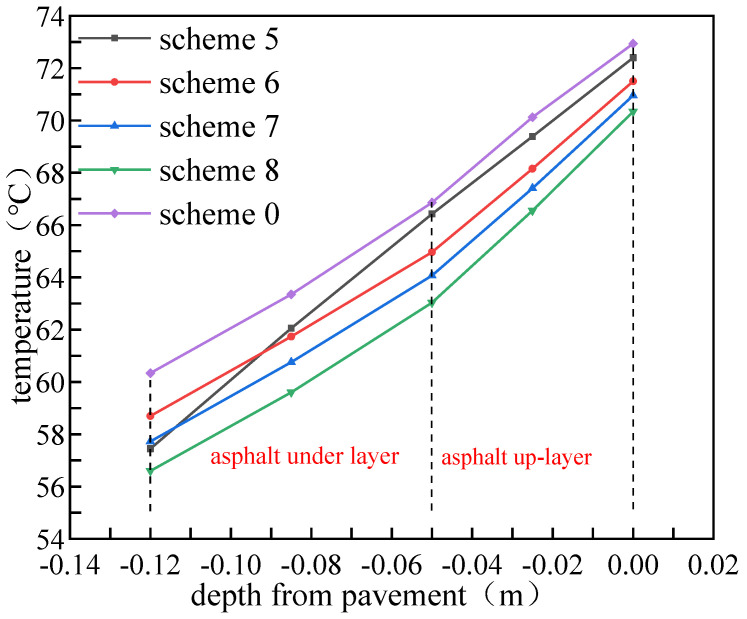
Temperature distributions of the asphalt layer with different PCM mixing ratios on July 15.

**Figure 10:**
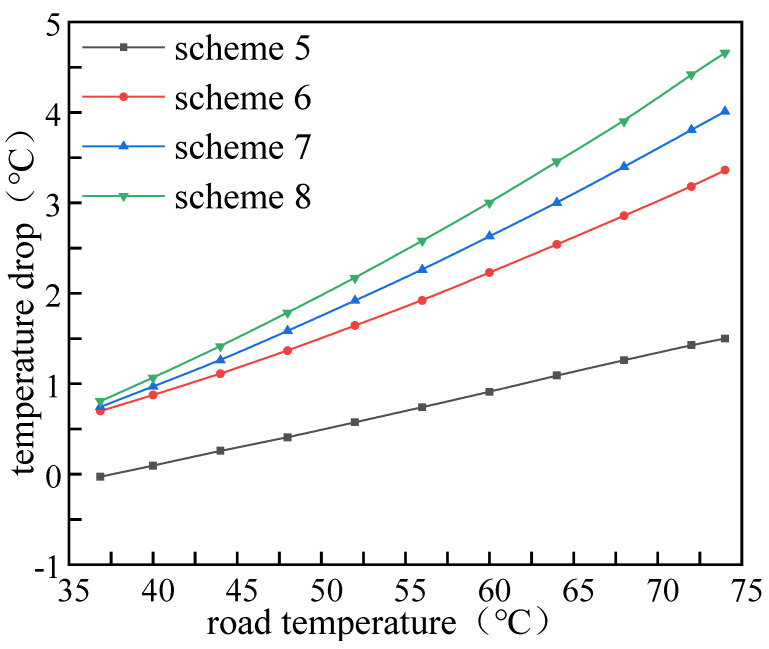
Temperature drop with different PCM mixing ratios.

The volume mixing ratio of PCM determines the thermal performance of the asphalt pavement. The temperature distribution of the asphalt layer on 15 July and temperature drop (based on Scheme 0) of the pavement with different PCM mixing ratios are shown in Figure 9 and Figure 10. It can be seen that the cooling effect of PCM is positively correlated to its volume mixing ratio. With the mixing ratio increasing from 5% to 20%, the pavement temperature decreased from 72.94 °C to 70.34 °C. In addition, the temperature drop also increased with the increase of PCM blending ratio, especially for the high pavement temperature condition. When the initial pavement temperature reached 75 °C, the temperature drop increased from 1.49 °C to 4.66 °C as the PCM mixing ratio increased from 5% to 20%.

**Figure 11:**
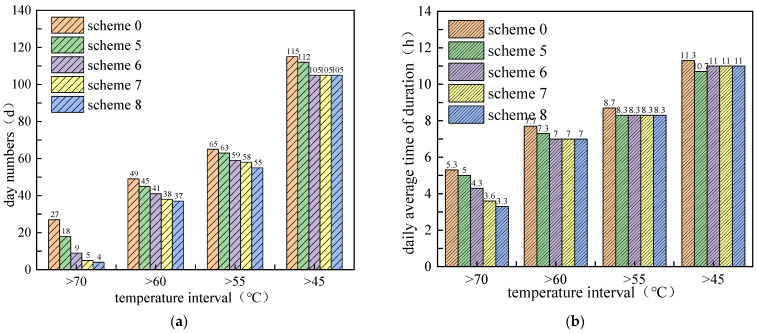
Duration times of daily maximum pavement temperature for different temperature ranges and PCM mixing ratio: (**a**) number of days; (**b**) daily average duration in hours.

Figure 11 shows the duration times of daily maximum pavement temperature for different temperature ranges and PCM mixing ratios. It can be seen that with the increase of the PCM mixing ratio, both the number of days and daily average duration decreased. Moreover, the cooling effect of PCM addition is more obvious in the high temperature range. With mixing ratio increases from 5% to 20%, the number of days and daily average duration decreased to 4 days and 3.3 h, respectively, in the temperature range of >70 °C. Compared with the non-PCM condition, the duration times of pavement high temperature were reduced by 23 days and 2 h. It also shows that in the lower temperature range, the cooling effect changed little when the PCM ratio exceeded 10%. However, it should be noted that although increasing the mixing ratio of PCM helps to improve the cooling effect, factors such as engineering cost and structural strength should also be considered when determining the mixing amount of PCM.

### 4.3. Cooling Efficiency of Different Mixing Positions

**Figure 12:**
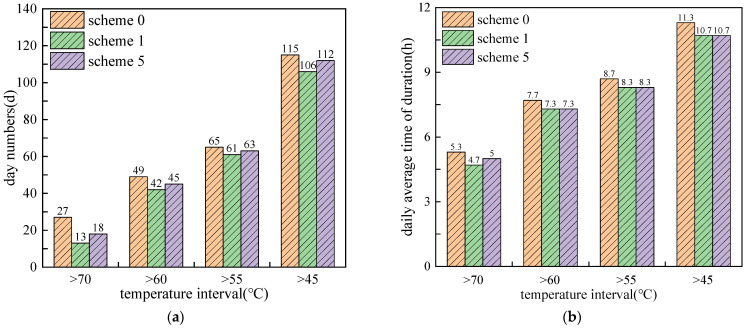
Duration times of daily maximum pavement temperature in different temperature ranges with 5% PCM mixing ratio: (**a**) number of days; (**b**) daily average duration in hours.

**Figure 13:**
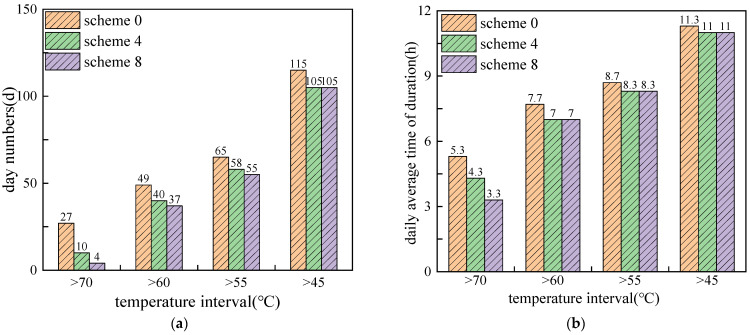
Duration times of daily maximum pavement temperature in different temperature ranges with 20% PCM mixing ratio: (**a**) number of days; (**b**) daily average duration in hours.

Figure 12 and Figure 13 show the duration times of daily maximum pavement temperature with different PCM mixing positions (overall asphalt layer PCM blended and asphalt upper layer PCM blended) and different PCM mixing ratio (5% and 20%). It can be seen that, with a volume mixing ratio of 5%, the asphalt upper layer PCM blended scheme has better performance than the overall asphalt layer PCM blended scheme. For scheme 1, the number of days of high temperature in the ranges of 45–55 °C, 55–60 °C, 60–70 °C, and above 70 °C decreased by 9 days, 4 days, 7 days and 14 days, respectively, and daily average duration in hours decreased by 0.6, 0.4, 0.4, and 0.6 h, respectively, which has larger value than observed for scheme 5.

In contrast, with a volume mixing ratio of 20%, the overall asphalt layer PCM blended scheme had better performance than the asphalt upper layer PCM blended scheme, especially in the high temperature range (>70 °C). The number of days for scheme 8 in the temperature ranges of 45–55 °C, 55–60 °C, 60–70 °C and above 70 °C decreased by 10 days, 10 days, 12 days and 23 days, respectively, and daily average duration hours decreased by 0.3, 0.4, 0.7 and 2.0 h, respectively. The above results indicate that the cooling effect of blending PCM near the high temperature pavement surface will be better improved at a small mixing ratio, whereas the performance resulting from mixing the entire asphalt layer with PCM will be effectively promoted by increasing the equivalent heat capacity of the system under the large mixing ratio.

## 5. Conclusions

In this study, a numerical model of the coupled heat transfer process of a typical monolithic subgrade of the G7 Expressway in the eastern Tianshan mountain area was developed. Three types of paraffin materials (OP55E, OP52E, OP47E) were mixed in a 4:3:3 volume ratio and blended into the asphalt upper layer and overall asphalt layer at the volume ratios of 5%, 10%, 15% and 20%. Then, the cooling effect of different PCM addition schemes was simulated and analyzed, and the frequency and duration times of the asphalt pavement high temperature operation status were also measured. The following conclusions were drawn:

(1) The addition of PCM in the asphalt layer can effectively reduce the frequency of pavement high temperature. The number of days and daily average duration of high temperature on the road surface are both reduced.
(2) The cooling effect is positively correlated to PCM volume mixing ratio and the temperature drop of the pavement also increases with the increase of PCM blending ratio. As the PCM mixing ratio increases from 5% to 20%, the 75 °C pavement can be cooled by 1.49 °C and 4.66 °C, and the number of days and daily duration of pavement temperatures over 70 °C decreased to 4 days and 3.3 h, respectively.
(3) The cooling effect of the asphalt upper layer PCM scheme is better at a small mixing ratio (5%), whereas the performance of the overall asphalt layer PCM blended scheme can be effectively promoted by increasing the equivalent heat capacity of the system under a large mixing ratio (20%).

## Figures and Tables

**Table 1:** Rutting risk level of different pavement temperature ranges [27].

Level of Rutting Risk	Temperature Range/°C	Main Features
I	45–55	Asphalt pavement is prone to aging under long-term high temperature, resulting in changes in macro performance and rutting risk.
II	55–60	Asphalt softens slowly and internal cohesion decreases. Under load, fluidity increases and dynamic stability decreases sharply.
III	60–70	The stiffness modulus decreases sharply, the asphalt softens rapidly, and the dynamic stability decreases sharply.

**Table 2:** Thermophysical parameters for different PCM.

Type	Melting Temperature Range/°C	Average Enthalpy of Phase Change (Kj/Kg)	Thermal Conductivity [W/(M∙K)]	Density (Kg/M^3^)	Volume Expansion Coefficient (%)
OP47E	41–48	17.85	0.2	825	12
OP52E	49–53	18.10	0.2	820	12.5
OP55E	51–57	19.57	0.2	825	14

**Table 3:** PCM addition schemes.

Addition Ratio	5%	10%	15%	20%
The asphalt upper layer (5 cm)	Scheme 1	Scheme 2	Scheme 3	Scheme 4
The overall asphalt layer (12 cm)	Scheme 5	Scheme 6	Scheme 7	Scheme 8
The traditional pavement	Scheme 0			

**Table 4:** Thermophysical parameters of solid materials.

Solid Layers	Thermal Parameters		
	$\rho \;(\mathrm{Kg}/M^{3})$	$C_{p}\;[J/(\mathrm{Kg}\cdot K)]$	λ [W/(M·K)]
Asphalt upper layer (5 cm)	2300	1000	1.3
Overall asphalt layer (12 cm)	2300	700	2.49
Water-stabilized gravel layer (53 cm)	2200	800	1.2
Subgrade filling soil layer	1980	1098	1.63
Silt layer (0.7 m)	1920	1500	1.26
Gravel layer (5.0 m)	2000	800	0.9
Sandy layer (24.3 m)	1700	730	2.69

## Data Availability

Not applicable.
